# Activity-based probes for dynamic characterisation of polysaccharide-degrading enzymes

**DOI:** 10.1042/BCJ20253060

**Published:** 2025-07-01

**Authors:** Isabelle B. Pickles, Thamy L.R. Corrêa, Herman S. Overkleeft, Gideon J. Davies

**Affiliations:** 1 York Structural Biology Laboratory, Department of Chemistry, The University of York YO10 5DD,U.K; 2 Department of Bio-organic Synthesis and Department of Medical Biochemistry, Leiden Institute of Chemistry, Leiden University, P.O. Box 9502, Leiden 2300 RA,Netherlands

**Keywords:** activity-based probes, biomass conversion, carbohydrate-active enzymes, enzyme inhibitors, glycobiology

## Abstract

Carbohydrate-active enzymes play essential roles in polysaccharide degradation, yet their biochemical characterisation remains challenging – especially in the face of rapidly expanding genomic and structural data. Standard annotations often overlook critical properties such as expression patterns, enzyme stability and substrate specificity, which are key to understanding function in biological and industrial contexts. Activity-based probes (ABPs) offer a direct solution by enabling selective detection of active enzymes within complex systems. This review focuses on ABPs for retaining glycosidases, tracing their development from early applications in medical diagnostics to emerging uses in biomass degradation. We examine recent advances in scaffold design – including fluorosugars, epoxides, aziridines and cyclic sulphates – and their utility in enzyme profiling, inhibitor discovery and biotechnology. Current ABPs remain limited: they cannot yet target inverting enzymes and other classes lacking nucleophilic residues, a gap that may be bridged through computational modelling and AI-guided probe development. Looking forward, integration of ABPs with enzyme engineering and design holds promise for unlocking new classes of biocatalysts tailored for industrial and biomedical use.

We are now in a post-genomic, post-AlphaFold world [1]. Genomic and metagenomic sequence data have outpaced our capacity for interpretation. High-quality three-dimensional structure predictions are widely available, and transcriptomic analyses can reveal mRNA expression levels for diverse proteins. Yet, despite these advances, methods for the rapid and dynamic biochemical characterisation of enzymes remain limited.

In the field of oligo- and polysaccharide degradation, challenges in biochemical characterisation are particularly acute. While the CAZy sequence-based classification (www.cazy.org [2]) has greatly advanced our ability to group enzymes into families, and sequence similarity networks (Figure 1) have helped refine sub-family distinctions (e.g. [3,4]), these tools often fall short of delivering functional insights. Genome annotations, though widely used, are frequently inaccurate or incomplete. Even when correct, they rarely capture essential properties such as enzyme expression dynamics, substrate specificity or stability – factors that are critical to biotechnological applications. Which enzymes are needed for efficient deconstruction of complex polysaccharides? How and when are they expressed? How do enzymes tolerate substrate branch points and substitutions or operate under harsh industrial milieu? Activity-based probes (ABPs; see reviews [5,6]) offer promising solutions to these pressing questions.

**Figure 1:**
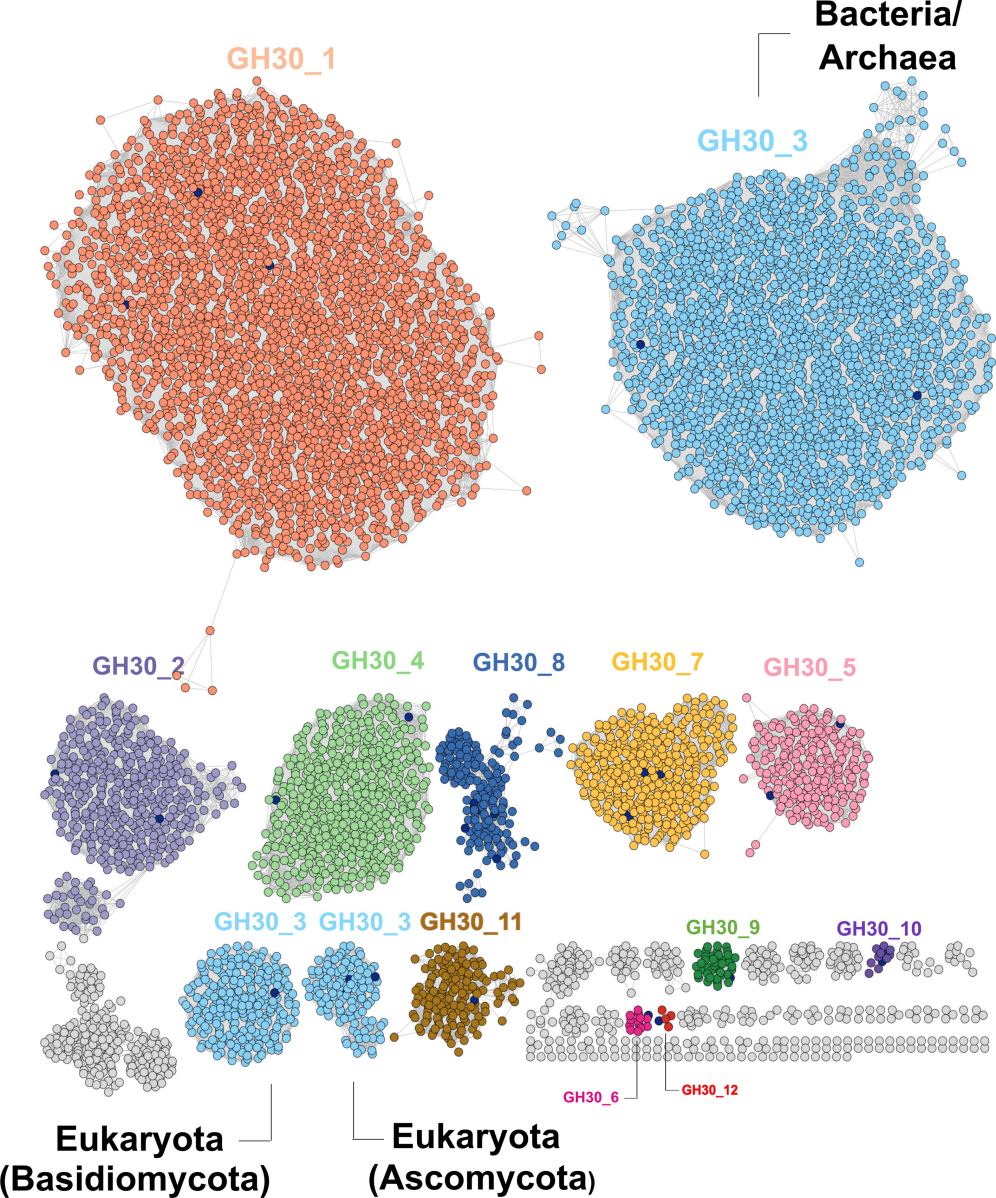
Sequence similarity network (SSN) reveals subfamily structure within GH30 glycoside hydrolases. A sequence similarity network (SSN) of 8,066 GH30 proteins was generated using the PFAM identifiers PF02055 and PF14587 via the EFI-EST tool (https://efi.igb.illinois.edu/efi-est/) and visualised in Cytoscape. Nodes represent sequences with ≥80% identity and are colour-coded by CAZy subfamily classification. Characterised sequences appear in dark blue; uncharacterised clusters – highlighting a need for the ABP approach – are grey. GH30_3 (light blue) distinctly separates Bacteria/Archaea and Eukaryota. Subfamilies span a range of activities including but not limited to β−glucosidase, xylosidase, fucosidase, glucuronoxylanase, galactosidase and glucuronidase (see https://www.cazy.org/GH30_activity.html). Edges denote pairwise similarities with e-values <10⁻⁵. The SSN was visualised using Cytoscape (https://cytoscape.org/).

## Historical development of ABPs

**Figure 2:**
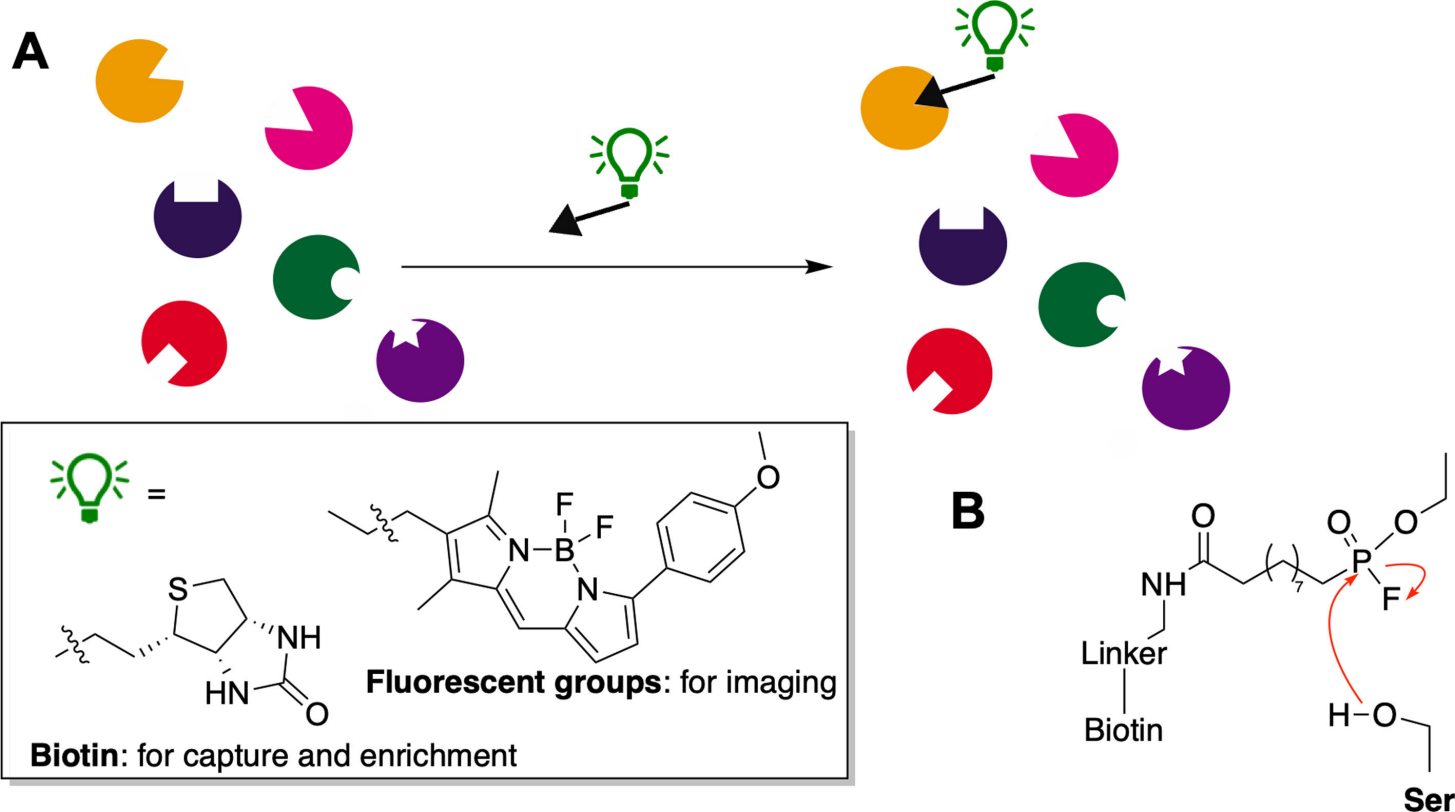
Activity-based probes (ABPs) and their historical context. (**A**) General structure of an ABP: active-site targeting group, recognition element and reporter tag. In two-step labelling the tag could be azide or alkyne for subsequent ‘click’ chemistry. (**B**) Cravatt’s fluorophosphonate probes, originally developed for serine hydrolases.

ABPs, first introduced by Cravatt [7], are composed of three main elements: an active-site targeting group, a recognition moiety to confer specificity (or broad reactivity), and a reporter tag – typically a fluorescent label for detection, or biotin for capture and enrichment (or an azide/alkyne for subsequent ‘click’ chemistry) (Figure 2A). Cravatt’s early probes were modelled after Sarin nerve agents and incorporated fluorophosphonates linked to biotin, which proved highly effective in profiling serine hydrolases (Figure 2B). This design established the foundational paradigm: a covalent electrophilic trap aligned with recognition and reporter modules. Building on this concept, the Bogyo group introduced epoxide electrophiles tailored for cysteine hydrolases [8], thereby expanding the probe’s applicability to enzymes with different nucleophilic centres.

**Figure 3:**
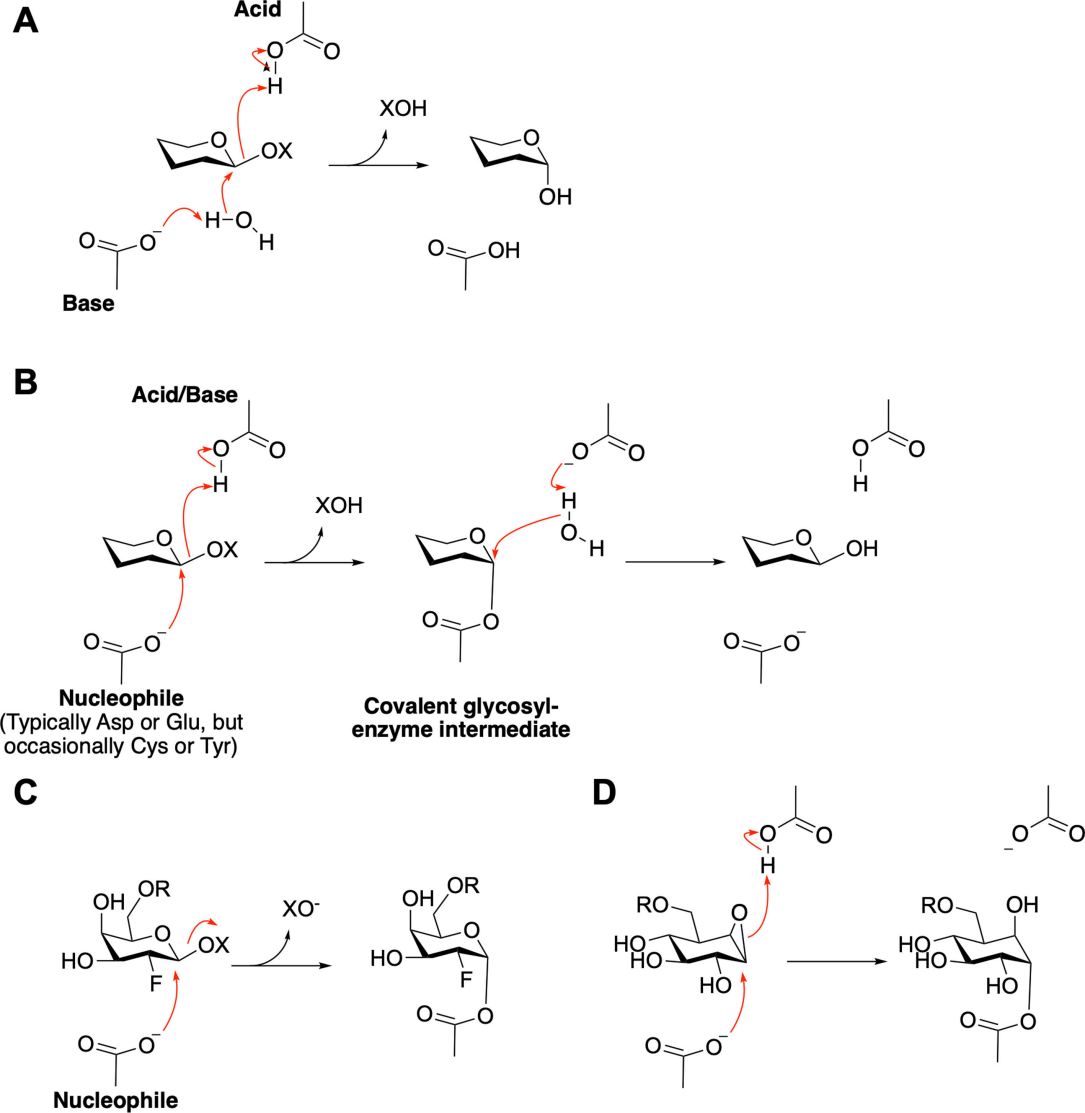
Glycosidase mechanism and design evolution of activity-based probes (ABPs) for covalent glycosidase labelling. (**A**) Generic glycosidase mechanism with inversion of anomeric configuration. (**B**) Generic glycosidase mechanism with net retention of anomeric configuration. Central to this mechanism is nucleophilic attack which is exploited by the ABP approaches described. Note this review will not cover NAD^+^ or neighbouring-group catalytic mechanisms. (**C**) Vocadlo and Bertozzi’s adaptation of Withers’ 2-fluoro sugars for glycosidase imaging. (**D**) Cyclophellitol-based ABP schematic for covalent labelling of retaining β-glucosidases.

Glycoside hydrolases involved in oligo- and polysaccharide degradation typically fall into two mechanistic categories, defined by the stereochemical outcome of hydrolysis (Figure 3A and B). Inverting enzymes catalyse a single displacement and thus invert the absolute configuration of the anomeric carbon following hydrolysis (Figure 3A). In retaining enzymes, a double displacement takes place via the formation and subsequent breakdown of a covalent glycosyl–enzyme intermediate (Figure 3B). Non-classical enzymes such as those using neighbouring group participation or NAD(H) are not considered further here. The classical retaining enzymes are prime targets for ABP approaches since they bear active centre nucleophilic groups that can be intercepted with appropriate electrophiles (Figure 3C and D). The majority of enzymes use enzymatic carboxylates (Asp and Glu) as nucleophiles (historically reviewed in Ref. [9]), although Tyr and Cys nucleophile enzymes also exist (and the latter also succumbs to ABP approaches [10,11]).

Progress in adapting ABP strategies to glycoside hydrolases was initially slow following Cravatt’s foundational work. Notably, Withers demonstrated the potential of 2- and 5-fluoroglycosides for trapping and identifying active-site nucleophiles in retaining glycosidases [12]. In 2004, Vocadlo and Bertozzi extended this approach by introducing 6-azido, 2-fluoro sugar derivatives, enabling one of the first recognisable applications of activity-based proteomics to β-galactosidases (Figure 3C) [13]. For some enzymes, the resulting 2/5-fluoro adducts are sufficiently long-lived to allow downstream *in vitro* or *in vivo* studies – examples of which are provided in [14–16]. However, in many cases, these mechanistic probes bind only transiently, limiting their utility. Effective use often required engineered enzyme variants, not a strategy applicable to activity-based profiling. Alternative probe designs, with long-lived adducts, are required for a more general ABP strategy.

Sugar epoxides – and their structural cousins, aziridines – emerged as elegant tools for covalently targeting the nucleophilic active sites of retaining glycosidases. However, the prototypical example, conduritol β-epoxide (CBE), proved problematic: due to its internal symmetry, CBE could interact with both α- and β-glycosidases, and in some cases, misidentified the nucleophilic residue [17]. As a result, CBE and related alkyl epoxides fell out of favour. In response, Withers proposed modifying these electrophiles by incorporating a C6 hydroxymethyl group – *such that it better resembles the natural glucoside substrate* [18]. This idea was realised in the natural product cyclophellitol, and in 2007, Madsen and colleagues developed a synthesis and demonstrated its binding to a β-glucosidase [19]. Recognising the synthetic route’s versatility, Overkleeft quickly adapted it to incorporate functional handles such as azides, fluorophores and biotin, thereby giving birth to the field of cyclophellitol-based activity-based proteomics for glycoside hydrolases (Figure 3D). With this foundation, ABPs quickly found medical applications.

## Applications in medical diagnostics

**Figure 4:**
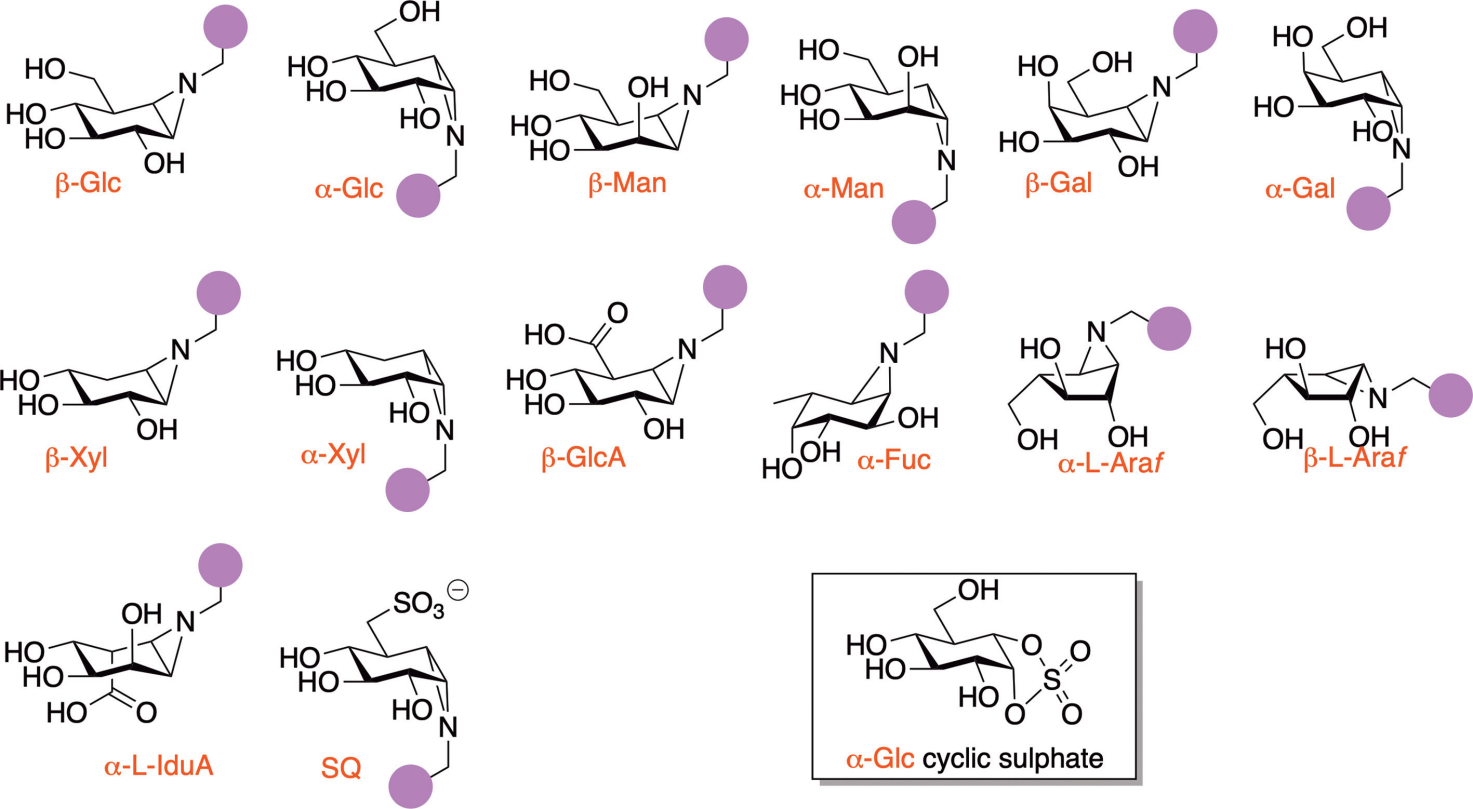
Monosaccharide ABPs featuring aziridines and cyclic sulphates for covalent glycosidase labelling. Representative aziridine-configured monosaccharide probes developed by Overkleeft and co-workers [20,21]. The boxed structure highlights an α-glucoside-configured cyclic sulphate ABP [23], used in antiviral applications targeting ER α-glucosidase II [22].

Overkleeft’s first work was to use modified cyclophellitols to image wildtype and disease-variant forms of the Gaucher disease β-glucosidase GBA. Since then, a wide array of monosaccharide sugar epoxides has been developed and deployed in the medical arena (Figure 4, reviewed in [20,21]). Sugar epoxides have been followed by sugar aziridines which allow functionalisation at the aziridine nitrogen and also by sugar cyclic sulphates [22], which often act as more reactive electrophilic traps – particularly for α-glycosidases. Specific cyclophellitol epoxides and aziridines have found use in the biomedical sphere, both for disease diagnostics and more recently also as leads for drug discovery. O6-tagged cyclophellitol (here glucopyranose numbering is used), which emulates the β-glucopyranose configuration, reports on the levels of active lysosomal glucosylceramidase (GBA1) in Gaucher disease samples [23]. Disease severity, including neuropathology, often depends on the specific point mutation involved, which can lead to partial or complete absence of active enzyme. This is reflected in the abundance of probe-tagged enzyme in patient tissue, visualised via fluorescence scanning of SDS-PAGE gels. Similarly, α−galactose-configured cyclophellitol aziridines can be used to monitor the lysosomal retaining α−galactosidase, GalA, which is deficient or absent in the genetic lysosomal storage disorder, Fabry disease [24]. Likewise, α-configured cyclophellitol aziridines reported on α−glucosidase, GAA, levels in Pompe samples [25].

## ABP-inspired enzyme inhibitors and therapeutics

Indeed, beyond their role as ABPs, it became increasingly clear – particularly in the medical context – that appropriately designed sugar epoxides, aziridines and cyclic phosphates could serve as potent enzyme *inhibitors*. Recent examples include the development of heparanase inhibitors and probes, notably in the anti-cancer context [26,27]. Tagged β-glucuronic acid-configured cyclophellitol aziridines label not only retaining β-exoglucuronidases but, uniquely among this class of monosaccharidic probes, also the human endoglucuronidase heparanase (HPSE) [27,28]. These compounds were subsequently adapted as potential anti-metastatic agents, motivated by the fact that HPSE is up-regulated in nearly all metastatic cancers, and that, despite decades of effort, no clinical inhibitors have yet emerged. Targeted anti-HPSE cyclophellitols have proven to be potent anti-cancer agents *in vitro* and in mouse models [26].

Readout of retaining β-exoglucuronidase activity has also been used for personalised diagnostic profiling of bacterial β-exoglucuronidases within individual-specific gut microbiomes [29]. Activity-based classification enables predictive assessment of drug toxicity in cases where liver-glucuronidated drugs are subsequently deglucuronidated in the intestine [29].

ABP designs have also been adapted for enzyme inhibition in the antiviral arena. As with most cyclophellitol aziridine configured probes, α-Glc configured cyclophellitol aziridines, epoxides, and cyclic sulphates have shown in-class selectivity for retaining α-glucosidases. Human cells constitutively express ER-α-glucosidase II (ER-II), a retaining enzyme, alongside ER-α-glucosidase I (ER-I; inverting). Together, these enzymes play key roles in ER quality control of nascent N-glycoproteins. Inhibitors of ER-glucosidases have gained attention as antiviral agents, particularly for viruses dependent on host N-glycosylation, with early-pandemic reviews highlighting their potential as anti-COVID therapies [30]. The α-Glc aziridine probe tags ER-II, but not GlcI, a result that inspired the design of epi-cyclophellitol cyclosulphate (Figure 2, boxed), a first-in-class ER-II-selective inhibitor that blocks SARS-CoV-2 replication in situ with comparable potency to current best-in-class competitive inhibitors [5]. Unlike these competitive inhibitors, however, which inhibit both ER-I and ER-II, the cyclophellitol probes allow precise dissection of GlcII’s role in viral infection.

These ABPs have also proven useful as readouts in complementary methods, including inhibitor or drug screening via fluorescence polarisation [31,32], and in competitive assays measuring EC₅₀ (recent examples include heparanase, β-glucuronidase [26,33] and α-mannosidase [34]). Versions with tuned-down reactivity can be used as conformational-based inhibitors and pharmacological chaperones for lysosomal α-glucosidase (GAA) in the context of Pompe disease [35]. In addition, ABPs have supported enzyme engineering efforts, including modular recombination strategies to evolve novel enzyme functions [36] (discussed further below).

## ABPs for the discovery and characterisation of biomass-degrading enzymes

Around 2018–2019, sugar epoxides, aziridines and cyclic sulphates began to be deployed as ABPs for retaining glycoside hydrolases in the context of biomass degradation – primarily to address the challenges outlined at the start of this review. In addition to the ‘Withers’ methodologies, described above, other methods were developed. These include tagged glycosylated quinone methides introduced by the Wright group and N-bromoacetyl sugars pioneered by Brumer and others (e.g. [37]).

**Figure 5:**
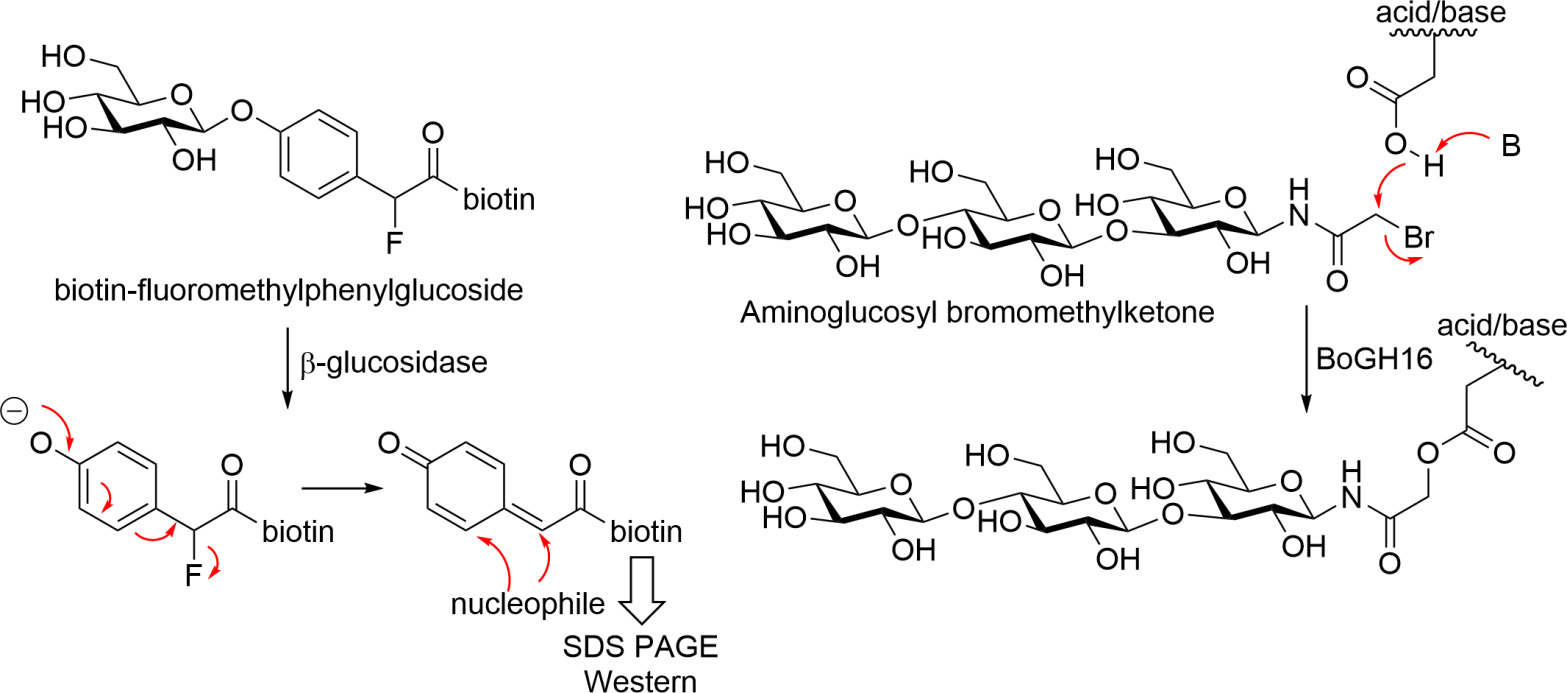
Alternative non-cyclophellitol ABPs used to target glycosidases. Examples of activity-based probes outside the cyclophellitol scaffold class, including quinone methide-releasing reagents and N-haloacetyl sugars, which have been applied to label glycosidases through diverse covalent mechanisms. ABPs, activity-based probes.

While the fluorosugars, like sugar epoxides and related compounds, demand an active centre nucleophile, the quinone methides and *N*-bromoacetyl sugars (at least in theory) do not. The former uses difluoromethylphenyl aglycones which generate a reactive quinone methide hunting for a nearby partner, while the *N*-bromo acetyl sugars are also somewhat promiscuous reacting with GH active centre acid/base carboxylic acid as well as other functional groups. The quinone methide strategy traces back to Lerner and Wong, who used biotin-tagged difluoromethylphenyl galactosides to screen for galactosidase activities in antibody libraries [38]. Capitalising on this strategy, Lo and coworkers designed biotin-fluoromethylphenyl glucoside (Figure 5) [39], and subsequently also a sialic acid analogue [40], as CAZyme-activated tagging reagents. Fluoride ion elimination occurs following enzyme-mediated hydrolysis of the glycosidic bond in this glucoside, generating the electrophilic quinone methide designed to react within the enzyme active site to form a stable covalent bond. Captured enzyme is then revealed by SDS PAGE and detection of biotin-protein by western blotting. The two Lo studies were done on recombinant, isolated enzyme and did not progress to ABPP on complex biological samples. However, more recently, Wright and coworkers revealed that a glucuronic acid-derived quinone methide probe enriches β-glucuronidases from extracts of commensal bacteria [41]. However, since labelling occurs only after enzymatic cleavage, and the reactive intermediate can diffuse out of the active site, off-target labelling remains a concern – especially in complex mixtures.

Withers addressed this by designing quinone methide-releasing imaging agents to localise CAZymes in cells and tissues [42]. Meanwhile, *N*-haloacetyl glycosides – known since 1971, when *N*-bromoacetyl β-D-galactopyranosylamine was used to label β-galactosidase [43] – have also seen renewed application. Brumer showed that a trisaccharidic aminoglucosyl bromomethylketone (Figure 4) could label BoGH16 endoglucanase via reaction with the catalytic acid–base residue rather than the nucleophile [44] (Figure 5. More recently, Wright and co-workers combined quinone methide, halomethylketone and fluorosugar strategies to create a suite of bioorthogonal ABPs for profiling cellulose-degrading enzymes in *Clostridium thermocellum* cellulosomes [45].

## Cyclophellitol ABPs for polysaccharide-degrading enzymes: xylanases and xylan-degrading enzymes

The first cyclophellitol-based ABPs for biomass degradation were developed for xylanases, using xylose- and xylobiose-based probes [46]. Xylan is the dominant polysaccharide in the hemicellulose fraction of the plant cell wall, closely associated with cellulose and often cross-linked with lignin [47]. The xylan backbone consists of β- [1,4]-linked xylose units and is decorated with acetyl (at O2 and/or O3), glucuronic acid (GlcA) or 4-O-methylglucuronic acid (Me-GlcA) (O2) and arabinofuranose (Ara*f*) (O2 and/or O3). These decorations follow no fixed pattern, varying by species, tissue and developmental stage [48]. This heterogeneity necessitates a suite of enzymes, each with distinct substrate preferences, to degrade xylan fully.

The initial ABP design was a xylobiosyl disaccharide aziridine, with a reporter group on the aziridine nitrogen – at the pseudo-‘reducing end’. A similar monosaccharide version was used to label retaining β-xylosidases. These probes revealed dynamic shifts in xylanase secretion depending on fungal growth substrate and provided ‘industrially relevant’ data on enzyme pH and thermal stability. Although the xylobiose probe did not differentiate between xylanase subtypes, competition with polymeric xylans revealed subtle differences in enzyme specificity hinting at the potential for future, more specific, probes.

This early work also highlighted some limitations. While GH10 xylanases were strongly labelled, GH11 enzymes showed little to no activity – possibly due to an incompatible conformational itinerary or insufficient probe length. Another issue was β-xylosidase-catalysed cleavage of the disaccharide probe to generate a monosaccharidase-active probe, complicating interpretation. This problem can be mitigated by using non-reducing-end-labelled epoxides (see for example cellulase and amylase work, below) or, where feasible, non-hydrolysable oligosaccharides – though the latter can be synthetically demanding [49].

Building on these insights, we are now developing cyclophellitol-derived ABPs specific for arabinoxylan- and glucuronoxylan-active xylanases. These new probes target xylan segments substituted with Ara*f* or GlcA, respectively. They not only broaden the current ABP toolkit but also offer a pathway to more precise substrate-specific profiling of xylan-degrading enzymes.

## Cyclophellitol ABPs for polysaccharide-degrading enzymes: cellulases

Cellulose, a linear polysaccharide composed of β-1,4-linked D-glucose, is the predominant structural carbohydrate in plant biomass. In 2020, De Boer and colleagues described the first cellulase-specific cyclophellitol-based ABP [50]. This probe was designed around a cellobiose disaccharide, featuring an epoxide as the electrophilic trap and a reporter group attached at the non-reducing end via the C4 position.

The system supported full inactivation kinetics (that is describing both *k*_inact_ and *K*_inact_ for the irreversible inactivation) and was validated through mass spectrometry and 3-D structural analysis of the *Humicola insolens* Cel7B. his disaccharide probe was used in parallel with a monosaccharide aziridine probe for β-glucosidases to monitor cellulase production in *Aspergillus niger* cultures grown on plant material. Notably, placement of the reporter group at the non-reducing end was key to preventing undesired hydrolysis by *exo*-acting β-glucosidases (as discussed previously).

In tandem with the xylanase probes defined previously, the cellulase-specific ABPs could then be applied to an array of basidomyces secretomes grown on maltose, wheat straw or aspen pulp. These experiments revealed a highly dynamic and complex pattern of enzyme secretion, shaped by both the carbon source and the organism’s growth stage [51], factors which would likely be lost in simple transcriptomics experiments (given the stability of the proteins, see sulfoquinovosidase example below). Similar findings were recently reported for the ascomycete *Parascedosporium putredinis* NO1, which tailors its enzyme production in response to different lignocellulosic substrates [52]. Our underlying proteomic methodologies were recently summarised in a dedicated review [53].

## Cyclophellitol ABPs for polysaccharide degrading enzymes: xyloglucanases

**Figure 6:**
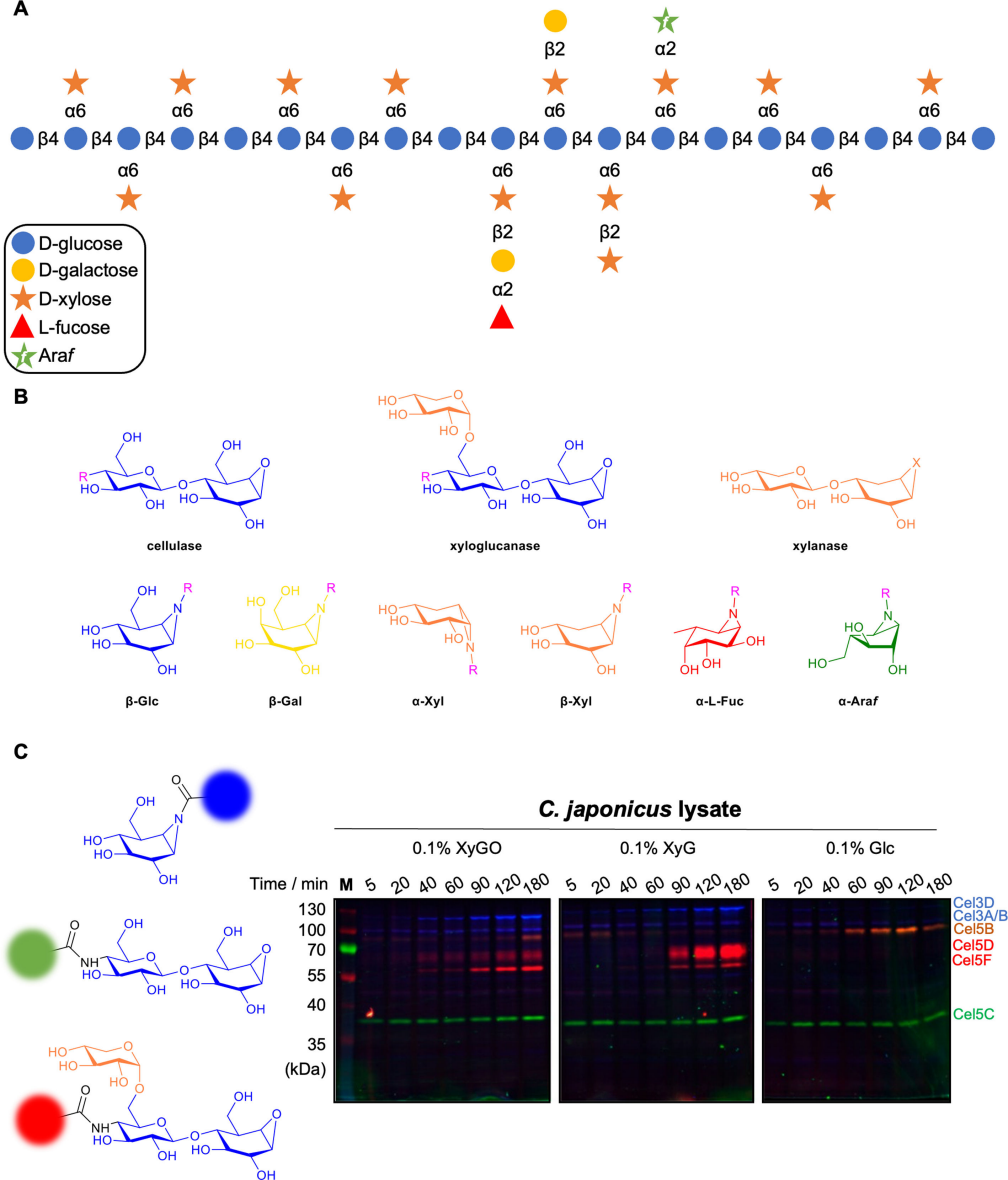
Activity-based probes (ABPs) enable structural and temporal mapping of xyloglucan degradation. (**A**) Representative xyloglucan structure: a β-1,4-glucan backbone with diverse side chains, including α-1,6-linked xylose and extended decorations (not all substitutions co-occur in nature, the scheme is for illustrative purposes only; see [54] for discussion of xyloglucan structure). (**B**) ABP panel used to target multiple retaining glycosidases involved in xyloglucan breakdown. (**C**) Multiplex ABP profiling of *Cellvibrio japonicus* enzyme expression over time, under different carbon sources (xyloglucan oligosaccharides, polymeric xyloglucan or glucose). Adapted from ref. [55].

Xyloglucan is another polymer found in the cell walls of higher plants. Xyloglucan is a highly branched polysaccharide composed of a β-1,4-glucose backbone, in which multiple glucose residues are substituted at the 6-position by xylosyl groups. These xylosyl residues are further modified with (1,2)-galactose, L-(1,2)-fucose, or, less frequently, additional xylosyl groups (Figure 6). The precise substitution pattern varies across species and tissues, contributing to the structural diversity observed [54,56].

Xyloglucanases – enzymes that degrade this complex polysaccharide – are distributed across several CAZy families, including GH5, GH9, GH12, GH16, GH44 and GH74. A trisaccharidic xyloglucan-configured cyclophellitol ABP, bearing an orthogonal fluorophore at the non-reducing end, was developed to probe these enzymes. This ABP was evaluated in a multiplex assay alongside cellulase-targeting cyclophellitol aziridines, enabling simultaneous analysis of multiple enzymes [55].

This ‘multiplex’ assay successfully identified a xyloglucanase-specific signal: a ~65 kDa band observed under xyloglucan conditions, later confirmed by proteomics as Cel5D – a protein previously annotated as a cellulase. Due to its selectivity, the ABP enabled precise monitoring of time- and substrate-dependent production of xyloglucanases by *C. japonicus*.

Interestingly, under arabinoxylan growth conditions, a cross-reactive band (~70 kDa) was detected with both ABP-XyG and ABP-Cel probes. Proteomics revealed this to be Cel5B, a cellulose-active enzyme with minor activity towards tamarind xyloglucan. These findings underscore the power of ABPs in dissecting complex enzymatic interactions and revealing substrate-specific roles within complex biological samples and secretomes [55].

## Cyclophellitol ABPs for polysaccharide degrading enzymes: amylases

**Figure 7:**
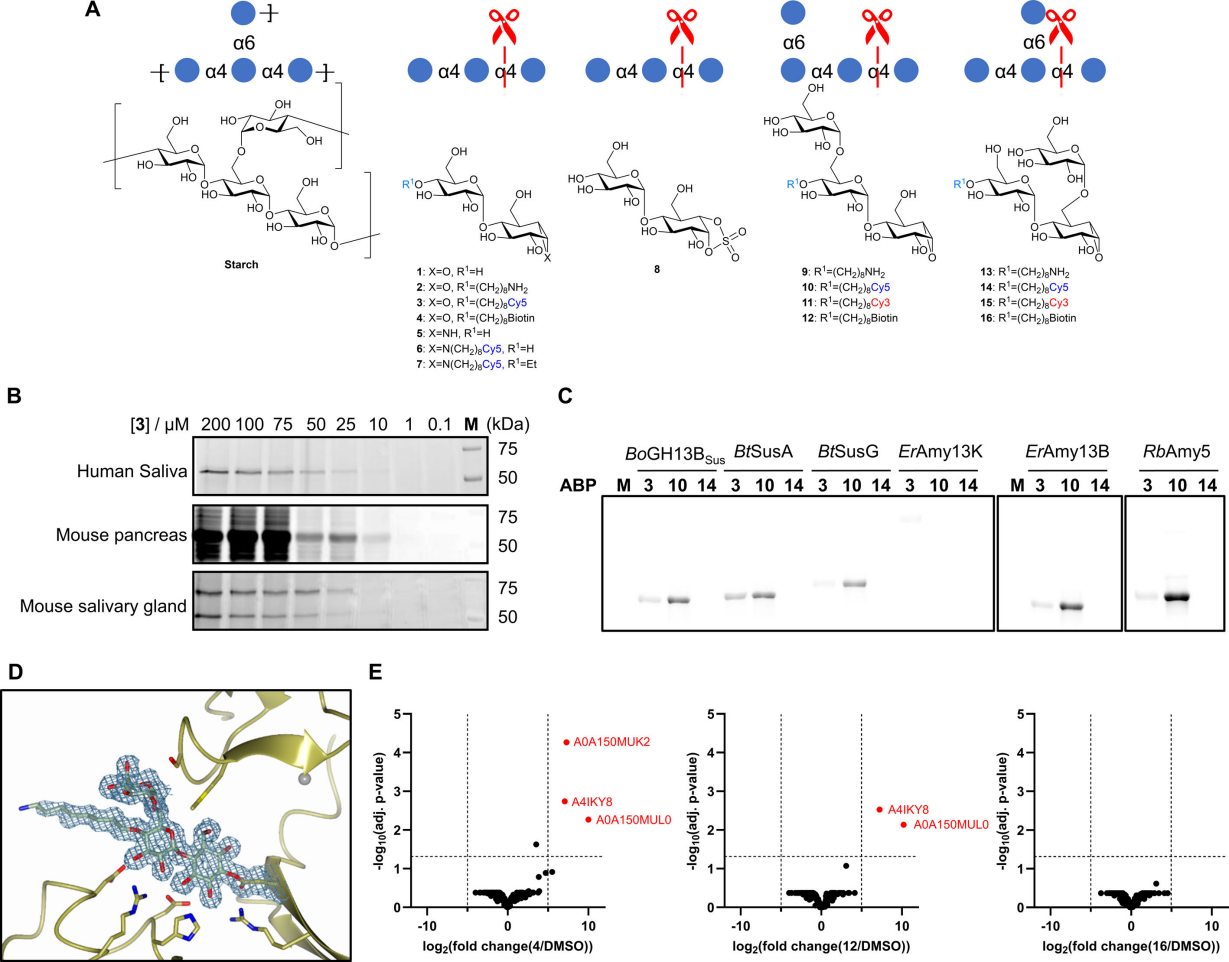
Probing α-amylase specificity and discovery using a modular activity-based probe (ABP) toolbox. (**A**) Structures of starch and α-amylase-targeted ABPs. (**B**) Labelling of α-amylases in complex biological samples with probe **3**. Adapted from [57]. (**C**) Substrate preferences of human gut α-amylases revealed by probes **3**, **10** and **14**. (**D**) Active site covalent binding of inhibitor **9** to *Ruminococcus bromii* amylase RbAmy5. (**E**) Discovery of novel α-amylases from compost using pull-down assays; volcano plots highlight proteins significantly enriched by probes **4**, **12** and **16**. C–E adapted from [58].

Starch is a highly heterogeneous polysaccharide composed of α-1,4-linked D-glucose units with irregular α-1,6 branch points (Figure 7). As the primary carbohydrate source in human and animal diets, it is of significant industrial relevance. Breakdown of the α-1,4 linkages in starch is catalysed by GH family 13 α-amylases, enzymes which are widely exploited in industrial starch processing as enzymatic alternatives to chemical hydrolysis. Many fungal and bacterial α-amylases are currently applied in this industrial processing, most commonly those from *Aspergillus oryzae* and *Bacillus licheniformis*. However, identifying novel enzymes with enhanced substrate specificity or tolerance to industrial conditions remains a slow and labour-intensive process.

A major advance came in 2016, when Carner *et al*., reported a maltobiose-configured epi-cyclophellitol inhibitor, synthesised via chemoenzymatic methods [59]. Time-dependent inhibition was observed, as well as evidence of a covalent reaction at the nucleophilic aspartate of human pancreatic α-amylase (HPA) in the x-ray crystal structure.

Building on this, Chen *et al*., (2021) developed a panel of α-amylase-targeting probes with varied electrophilic warheads [57]. Carner’s probe was adapted by attaching an alkyl amine to the O4′ position of the non-reducing sugar, which was then coupled to Cy5 or biotin NHS esters – or capped with an ethyl group to prevent *exo*-acting α-glucosidase degradation. In addition, aziridine variants with similar modifications at the aziridine nitrogen (with or without the O4′ cap) were synthesised, along with a cyclosulphate version. Interestingly, although aziridines generally outperform epoxides against α-glucosidases [25], the aziridine maltobiose showed reduced inhibition of TAKA α-amylase compared with the ‘uncapped’ epoxide probe.

The expected covalent reaction with all three warheads was demonstrated by protein structure determination. In-gel ABPP with the Cy5 tagged probes demonstrated much more potent labelling of human saliva α-amylase by the O4′-linked epoxide probe than both the ‘capped’ and ‘uncapped’ aziridines. The maltobiose *epi*-cylcophellitol probe was used to detect α-amlyases in human saliva and mouse pancreas and salivary gland, as well as fungal *Aspergillus nidulans* secretomes. Furthermore, ABPP performed under varied pH and temperature conditions enabled rapid profiling of enzymatic optima. Pull-down experiments with biotinylated probes followed by proteomic analysis confirmed successful identification of the labelled amylases in both human and fungal samples.

Building upon this success, we recently reported the design of a new toolbox of α−amylase covalent inhibitors and probes with varying α−1,6 branch point mimics [58]. These compounds are derived from the original maltobiose *epi*-cylophellitol core but incorporate α−1,6 branches in either the ‘-1’ or ‘-2’ subsite positions.

The probes were applied to a panel of human gut bacterial α-amylases and demonstrated a strong preference, in many cases, for the −2 branched variant. This was observed in both ABPP gels using purified enzymes and in lysates from *Bacteroides thetaiotaomicron*, as well as in biotin-based pull-down proteomic experiments. Interestingly, probe binding varied by warhead: some enzymes showed a preference for the unbranched aziridine probe, others favoured the epoxide version, and a few exhibited no clear preference. These findings illustrate how the positioning of reporter groups can significantly affect probe–enzyme interactions, even among closely related α-amylases.

The same toolbox was used to profile a selection of industrially relevant α−amylases for pH, temperature and calcium dependence, as well as for branch point accommodation. Notably, a calcium-independent α-amylase from *Bacillus* sp. KSM-K38 was shown, via in-gel ABPP and structural biology, to prefer the −2 branched mimic – an unexpected property not previously reported.

To explore discovery potential in environmental samples, the three biotinylated probes (unbranched, −1 branched and −2 branched) were used in pull-down assays with a commercially available compost sample. Three α-amylases were captured by the unbranched probe, two by the −2 branched probe and none by the −1 branched variant – consistent with the known inability of α-amylases to accommodate a branch at that subsite. One of the unbranched-probe-specific enzymes had been annotated as a neopullulanase (A0A150MUK2 from *Parageobacillus toebii*), but the probe data allowed for more precise functional annotation: this enzyme was identified as a true α-amylase that lacks tolerance for branching at the −2 subsite. AlphaFold models of the three uncharacterised enzymes revealed structural features consistent with their observed substrate preferences, reinforcing the use of ABPs in functional annotation.

## Mannan degradation

Linear mannans consist of a β-1,4-linked backbone, which in the case of galactomannans, is further substituted with α-1,6 galactose residues. An alternating β−1,4 linked backbone of glucose and mannose defines glucomannans (which may also be substituted in galactoglucomannans).

Degradation of these polymers requires a combination of enzymes: mannanases or glucomannanases to cleave the main chain, β−mannosidases to act at the non-reducing ends, and various accessory enzymes to remove side chains. The design of ABPs for these enzymes presents conformational challenges given the B_2,5_ transition-state preference for the mannosidase reaction ([60,61]). Nevertheless, prior work [34] on α−mannosidases that targeting these enzymes with ABPs was a viable strategy.

To initiate probes for mannan degradation, a series of mannose-based ABPs was prepared with both epoxide and aziridine warheads [62]. These probes successfully labelled family GH2, GH5 and GH164 β-mannosidases and could be used to label mammalian β-mannosidases from mouse kidney extracts. Further work will no doubt extend to disaccharide inhibitors for β-mannanases and diverse glucomannanases.

## Arabinoside removal

l-Arabinofuranose is a common side chain in hemicellulosic polysaccharides such as arabinan, arabinoxylan and arabinogalactan. Its removal by α-l-arabinofuranosidases alleviates steric hindrance, allowing backbone-degrading enzymes better access to their substrates [63].

The first l-arabinofuranose-mimicking scaffolds were developed using a range of electrophilic traps informed by ligand complexes of α-l-arabinofuranosidases from the GH51 and GH54 families. Contrary to expectations, the aziridine inhibitor better mimics the natural glycosyl intermediate than the cyclic sulphate. Surprisingly, aziridine-based inhibitors provided a better mimic of the glycosyl-enzyme intermediate than cyclic sulphates. Structural analysis confirmed that aziridine-based probes formed covalent complexes with bacterial GH51 and GH54 enzymes without introducing the rearrangements or steric clashes observed in GH51–cyclic sulphate complexes.

When applied to *Aspergillus niger* cultures grown on arabinan, these probes were a little non-specific, although there is a notable enrichment of GH51 and GH54 enzymes when using the biotinylated version of the aziridine ABP, highlighting its utility for selectively identifying target enzymes within complex mixtures [64]. Consistent with broader trends in CAZy family behaviour, GH51 enzymes from both bacterial and fungal origins share a conserved catalytic site and follow the same catalytic mechanism. This was further supported by structural studies of fungal GH51 complexes with aziridine- and cyclic sulphate-based l-arabinofuranose probes, which behaved similarly [65].

β-l-Arabinofuranosides are rare sugar residues found in plant glycans such as arabinans, arabinogalactans and glycosylated hydroxyproline. Their degradation is catalysed by members of CAZy families GH127 and GH146, which have been proposed to operate via an unusual retaining mechanism involving a Zn²^+^-co-ordinated cysteine nucleophile.

Providing experimental support for this mechanism proved challenging, particularly beyond site-directed mutagenesis. A key energetic question emerged: how can deglycosylation proceed from a cysteine adduct, which is typically quite stable? This question was addressed using epoxide-based inhibitors, which, through kinetic analysis and mass spectrometry, demonstrated that the active-site cysteine functions as a nucleophile in both GH127 and GH146 [11].

Three-dimensional analysis of the adducts – notably with GH146 reacting at the ‘wrong’ epoxide carbon – enabled molecular dynamics simulations that probed the reaction co-ordinate in detail. These studies highlighted the complex energetics and the critical role of Zn²^+^ in tuning the cysteine’s reactivity to facilitate deglycosylation. The work also demonstrated how ABP adducts can serve as a foundation for further computational modelling.

Building on this, more reactive aziridine-based ABPs were synthesised and showed strong labelling of both GH127 and GH146 enzymes [10]. Structural analysis of the GH146–aziridine complex confirmed that the anomeric-mimicking C1 of the ligand reacts with the nucleophile at distances consistent with classical retaining glycosidases. These findings suggest that the enhanced reactivity of aziridines may reflect the match between the enzyme’s pH optimum and the pKa of the inhibitor class [10].

## ABPs for sulfoquinovose degrading systems

Sulfoquinovose (SQ) is a sulfosugar most commonly found as the head group of the sulfolipid sulfoquinovosyl diacylglycerol (SQDG), present in various plants, algae and bacteria [66]. Remarkably, the global turnover of SQ is estimated to rival that of the sulphur-containing amino acids cysteine and methionine, underscoring its importance in the sulphur cycle.

SQDG is hydrolysed to give SQ by classical (NAD-dependent enzymes [67] are not considered in this review) retaining GH31 enzymes sulfoquinovosidases (SQases) [68]. In 2024, Li and colleagues reported the first ABPs and inhibitors targeting GH31 SQases, based on epi-cyclophellitol aziridines modified with an O6-sulphate group and tagged via the aziridine nitrogen [69]. These probes enabled the detection of five representative SQases through in-gel fluorescence, intact mass spectrometry and structural biology.

Expression of SQases was confirmed in *Escherichia coli* and *Pseudomonas putida* when grown on SQ but not glucose, demonstrating substrate-induced expression. A biotinylated probe enabled pull-down proteomics to identify the labelled enzyme, while the Cy5-tagged version was used to monitor activity across the different growth stages of *Pseudomonas putida*. Interestingly, SQase activity peaked between early and late logarithmic growth before falling in stationary phase.

This dynamic pattern contrasted sharply with mRNA expression, which declined rapidly during logarithmic growth. These findings suggest that SQase protein persists longer than its transcript, emphasising the value of ABPP for directly measuring enzymatic activity *in vivo* – complementing transcriptomic analysis in order to see the whole picture of enzyme activity in a living system.

## ABPs and biofilm degradation

Beyond biomass, ABPs are now being deployed to probe at biofilm degrading and modifying enzymes. A prominent example is the Psl biofilm polymer, a repeating polymer of 2-*O*-(1,2-α-D-Man*p*)-1,3-β-D-Man*p*-1,3-β-D-Man*p*-1,3-α-L-Rham*p*-1,3-β-D-Glc*p*. Psl contributes to the structure and protection of biofilms formed by the opportunistic pathogen *Pseudomonas aeruginosa*.

The gene cluster responsible for Psl biosynthesis includes *pslG*, encoding a glycoside hydrolase. It has been proposed that processing of Psl by this enzyme may regulate biofilm formation and could present a target for antimicrobial strategies. *PslG* was initially annotated as an endo-β-mannosidase from GH39 [70]. Recently, however, Ruijgrok *et al*. have reported the development of a suite of activity-based probes based on different cleavage sites of the Psl substrate, revealing PslG is in fact an endo-β-glucosidase [71].

This reclassification highlights the potential of ABPs to refine enzyme annotations and uncover hidden enzymatic functions in complex biological contexts like biofilms. It will be exciting to see how these tools are further applied to explore biofilm biology and identify novel targets for therapeutic intervention.

## Limitations, drawbacks and future challenges

Despite the progress made across substrate classes and enzyme families, several key limitations and opportunities remain for ABP development. *Endo*-acting enzymes often require longer probes to match extended substrate binding sites. For example, while the α-amylase probe toolbox has enabled detailed specificity profiling, even greater resolution could be achieved with longer, more branched substrates. But longer probes come with drawbacks, notably they are themselves substrates and can be degraded, not only by the very enzymes they are designed to target, but also other cellular glycosidases. This was observed with the original xylanase work [46]. Non-hydrolysable versions of ABPs can, indeed, have, been made [49], but their synthesis is often complex and not easily scalable – posing a barrier to broader dissemination and adoption across research laboratories.

Another key challenge is the optimal placement of reporter groups such as biotin or fluorescent tags. While attachment at the non-reducing end can prevent hydrolysis by exoglycosidases, probe behaviour is also influenced by enzyme subsite architecture. Reducing-end and non-reducing-end probes may show different reactivities. Such observations do open the possibility to engineering additional ‘positive subsite’ recognition through incorporation of aziridine linked sugars; such compounds could allow for differentiation of, for example, α-1,2, α-1,3, α-1,4, α-1,6 glucosidases as well as identifying enzymes with specificity for other sugars in +1 subsite, for example.

A significant limitation of current ABPs is the absence of effective probes for inverting glycosidases, as well as retaining enzymes that utilise neighbouring group participation mechanisms. These enzyme classes lack an enzymatic nucleophile, so facile electrophilic traps are not appropriate. Conserved catalytic acids and bases in these enzyme classes provide a possible reactive group, but cunning design, perhaps aided by computational modelling or artificial intelligence, will be required to provide suitable probes (and related covalent inhibitors for medical application). Oddly, the *N*-bromo acetyl sugars have been observed to react with the catalytic acid/base of retaining xyloglucanases, but do not react with the equivalent residue of inverting enzymes, suggesting more fundamental reactivity work is required. It is possible that artificial intelligence approaches to ligand binding, already deployed successfully in drug design (for example Ref. [72]), may soon play a central role here.

Glycosyltransferases (GTs), which also lack a nucleophile, are another class ripe for ABP development. These nucleotide- or lipid-sugar-dependent enzymes are pivotal to glycan biosynthesis. Beyond theoretical designs alone, a more integrated approach – combining chemical insight with computational modelling – is likely to yield reactive scaffolds suitable for the inhibition and imaging of glycosyltransferases. AI approaches will increasingly become relevant here. Indeed, the specific inhibition of glycosyltransferases remains the holy grail of glycobiology if we are ever to have a fully integrated understanding of glycoscience and exploit the full potential of GT inhibition in the pharmacological space.

Key to the uptake of ABPs will be the scalability of their synthesis, the selection of targets that match challenges in medical and cellular biology and increasingly industrial biotechnology. Applying them in innovative ways for enzyme discovery and characterisation, deploying them on complex biological samples, such as secretomes and industrial bioreactors. With powerful computational approaches, ABPs will reach other enzyme classes. Indeed, the palette of new carbohydrate active enzymes is evolving [73]. The exciting use of fluorescent cyclophellitols for computational enzyme design, exemplified by Fleischmann’s work on xylanase [36], offers a glimpse into the future: ABPs applied for the design and optimisation of industrially stable enzymes, bespoke enzyme classes and novel functionalities. The fusion of ABPs with computational enzyme design not only promises enhanced enzyme discovery but also heralds a new era of biocatalysts, tailored for industrial and biomedical challenges yet to be imagined.

## Abbreviations

ABPs: activity-based probes
CBE: conduritol β-epoxide
HPA: human pancreatic α-amylase
HPSE: human endoglucuronidase heparanase

## References

[1] Jumper J. Evans R. Pritzel A. Green T. Figurnov M. Ronneberger O. et al 2021 Highly accurate protein structure prediction with AlphaFold Nature 596 583 589 10.1038/s41586-021-03819-2 34265844 PMC8371605

[2] Drula E. Garron M.L. Dogan S. Lombard V. Henrissat B. Terrapon N 2022 The carbohydrate-active enzyme database: functions and literature Nucleic Acids Res. 50 D571 D577 10.1093/nar/gkab1045 34850161 PMC8728194

[3] Aspeborg H. Coutinho P.M. Wang Y. Brumer H. 3rd Henrissat B 2012 Evolution, substrate specificity and subfamily classification of glycoside hydrolase family 5 (GH5) BMC Evol. Biol. 12 186 10.1186/1471-2148-12-186 22992189 PMC3526467

[4] Viborg A.H. Terrapon N. Lombard V. Michel G. Czjzek M. Henrissat B. et al 2019 A subfamily roadmap of the evolutionarily diverse glycoside hydrolase family 16 (GH16) J. Biol. Chem. 294 15973 15986 10.1074/jbc.RA119.010619 31501245 PMC6827312

[5] Thaler M. Ofman T.P. Kok K. Heming J.J.A. Moran E. Pickles I. et al 2024 *Epi*-cyclophellitol cyclosulfate, a mechanism-based endoplasmic reticulum α-glucosidase ii inhibitor, blocks replication of sars-cov-2 and other coronaviruses ACS Cent. Sci. 10 1594 1608 10.1021/acscentsci.4c00506 39220688 PMC11363342

[6] Willems L.I. van der Linden W.A. Li N. Li K.Y. Liu N. Hoogendoorn S . et al 2011 Bioorthogonal chemistry: applications in activity-based protein profiling Acc. Chem. Res. 44 718 729 10.1021/ar200125k 21797256

[7] Liu Y. Patricelli M.P. Cravatt B.F 1999 Activity-based protein profiling: the serine hydrolases Proc. Natl. Acad. Sci. U.S.A. 96 14694 14699 10.1073/pnas.96.26.14694 10611275 PMC24710

[8] Greenbaum D. Medzihradszky K.F. Burlingame A. Bogyo M 2000 Epoxide electrophiles as activity-dependent cysteine protease profiling and discovery tools Chem. Biol. 7 569 581 10.1016/S1074-5521(00)00014-4 11048948

[9] Davies G. Henrissat B 1995 Structures and mechanisms of glycosyl hydrolases Structure 3 853 859 10.1016/S0969-2126(01)00220-9 8535779

[10] Borlandelli V. Offen W.A. Moroz O. Nin-Hill A. McGregor N. Binkhorst L. et al 2023 β-l-*Arabino*furano-cyclitol aziridines are covalent broad-spectrum inhibitors and activity-based probes for retaining β-L-arabinofuranosidases ACS Chem. Biol. 18 2564 2573 10.1021/acschembio.3c00558 38051515 PMC10728902

[11] McGregor N.G.S. Coines J. Borlandelli V. Amaki S. Artola M. Nin‐Hill A. et al 2021 Cysteine nucleophiles in glycosidase catalysis: application of a covalent β‐ l‐ Arabinofuranosidase inhibitor Angew. Chem. Int. Ed. 133 5818 5822 10.1002/ange.202013920 33528085

[12] Withers S.G. Street I.P. Bird P. Dolphin D.H 1987 2-Deoxy-2-fluoroglucosides: a novel class of mechanism-based glucosidase inhibitors J. Am. Chem. Soc. 109 7530 7531 10.1021/ja00258a047

[13] Vocadlo D.J. Bertozzi C.R 2004 A strategy for functional proteomic analysis of glycosidase activity from cell lysates Angew. Chem. Int. Ed. 43 5338 5342 10.1002/anie.200454235 15468183

[14] Hekmat O. He S. Warren R.A.J. Withers S.G 2008 A mechanism-based ICAT Strategy for Comparing Relative Expression and Activity Levels of Glycosidases in Biological Systems J. Proteome Res. 7 3282 3292 10.1021/pr7008302 18563928

[15] Hekmat O. Kim Y.W. Williams S.J. He S. Withers S.G 2005 Active-site peptide “fingerprinting” of glycosidases in complex mixtures by mass spectrometry. Discovery of a novel retaining beta-1,4-glycanase in Cellulomonas fimi J. Biol. Chem. 280 35126 35135 10.1074/jbc.M508434200 16085650

[16] Williams S.J. Hekmat O. Withers S.G 2006 Synthesis and testing of mechanism‐based protein‐profiling probes for retaining endo‐glycosidases Chembiochem 7 116 124 10.1002/cbic.200500279 16397879

[17] Rempel B.P. Withers S.G 2008 Covalent inhibitors of glycosidases and their applications in biochemistry and biology Glycobiology 18 570 586 10.1093/glycob/cwn041 18499865

[18] Caron G. Withers S.G 1989 Conduritol aziridine: a new mechanism-based glucosidase inactivator Biochem. Biophys. Res. Commun. 163 495 499 10.1016/0006-291X(89)92164-5 2673241

[19] Gloster T.M. Madsen R. Davies G.J 2007 Structural basis for cyclophellitol inhibition of a β-glucosidase Org. Biomol. Chem 5 444 446 10.1039/B616590G 17252125

[20] Artola M. Aerts J.M.F.G. van der Marel G.A. Rovira C. Codée J.D.C. Davies G.J . et al 2024 From Mechanism-Based Retaining Glycosidase Inhibitors to Activity-Based Glycosidase Profiling J. Am. Chem. Soc. 146 24729 24741 10.1021/jacs.4c08840 39213505 PMC11403624

[21] Wu L. Armstrong Z. Schröder S.P. Boer C. d. Artola M. Aerts J.M.F.G . et al 2019 An overview of activity-based probes for glycosidases Curr. Opin. Chem. Biol. 53 25 36 10.1016/j.cbpa.2019.05.030 31419756

[22] Artola M. Wu L. Ferraz M.J. Kuo C.L. Raich L. Breen I.Z. et al 2017 1,6-Cyclophellitol cyclosulfates: a new class of irreversible glycosidase inhibitor ACS Cent. Sci. 3 784 793 10.1021/acscentsci.7b00214 28776021 PMC5532717

[23] Witte M.D. Kallemeijn W.W. Aten J. Li K.Y. Strijland A. Donker-Koopman W.E. et al 2010 Ultrasensitive in situ visualization of active glucocerebrosidase molecules Nat. Chem. Biol. 6 907 913 10.1038/nchembio.466 21079602

[24] Willems L.I. Beenakker T.J.M. Murray B. Scheij S. Kallemeijn W.W. Boot R.G. et al 2014 Potent and selective activity-based probes for GH27 human retaining α-galactosidases J. Am. Chem. Soc. 136 11622 11625 10.1021/ja507040n 25105979

[25] Jiang J. Kuo C.L. Wu L. Franke C. Kallemeijn W.W. Florea B.I. et al 2016 Detection of active mammalian GH31 α-glucosidases in health and disease using in-class, broad-spectrum activity-based probes ACS Cent. Sci. 2 351 358 10.1021/acscentsci.6b00057 27280170 PMC4882745

[26] de Boer C. Armstrong Z. Lit V.A.J. Barash U. Ruijgrok G. Boyango I . et al 2022 Mechanism-based heparanase inhibitors reduce cancer metastasis in vivo Proc. Natl. Acad. Sci. U.S.A 119 e2203167119 10.1073/pnas.2203167119 35881786 PMC9351465

[27] Wu L. Jiang J. Jin Y. Kallemeijn W.W. Kuo C.L. Artola M. et al 2017 Activity-based probes for functional interrogation of retaining β-glucuronidases Nat. Chem. Biol. 13 867 873 10.1038/nchembio.2395 28581485

[28] Wu L. Viola C.M. Brzozowski A.M. Davies G.J 2015 Structural characterization of human heparanase reveals insights into substrate recognition Nat. Struct. Mol. Biol. 22 1016 1022 10.1038/nsmb.3136 26575439 PMC5008439

[29] Jariwala P.B. Pellock S.J. Goldfarb D. Cloer E.W. Artola M. Simpson J.B . et al 2020 Deciphering differential gut bacterial drug metabolism with activity-based protein profiling ACS Chem. Bio. 15 217 225 10.1021/acschembio.9b00788 31774274 PMC7321802

[30] Williams S.J. Goddard-Borger E.D 2020 α-glucosidase inhibitors as host-directed antiviral agents with potential for the treatment of COVID-19 Biochem. Soc. Trans. 48 1287 1295 10.1042/BST20200505 32510142

[31] Lahav D. Liu B. van den Berg R.J. van den Nieuwendijk A.M.C.H. Wennekes T. Ghisaidoobe A.T . et al 2017 A fluorescence polarization activity-based protein profiling assay in the discovery of potent, selective inhibitors for human nonlysosomal glucosylceramidase J. Am. Chem. Soc. 139 14192 14197 10.1021/jacs.7b07352 28937220 PMC5677758

[32] van der Gracht D. Rowland R.J. Roig-Zamboni V. Ferraz M.J. Geurink P.P. Aerts J.M.F.G . et al 2023 Fluorescence polarisation activity-based protein profiling for the identification of deoxynojirimycin-type inhibitors selective for lysosomal retaining alpha- and beta-glucosidases Chem. Sci. 14 9136 9144 10.1039/d3sc01021j 37655021 PMC10466331

[33] Chen Y. van den Nieuwendijk A.M.C.H. Wu L. Moran E. Skoulikopoulou F. Riet V. v . et al 2024 Molecular basis for inhibition of heparanases and β-glucuronidases by siastatin B J. Am. Chem. Soc. 146 125 133 10.1021/jacs.3c04162 38118176 PMC10785800

[34] Armstrong Z. Kuo C.L. Lahav D. Liu B. Johnson R. Beenakker T.J.M. et al 2020 Manno-*epi*-cyclophellitols enable activity-based protein profiling of human α-mannosidases and discovery of new golgi mannosidase II inhibitors J. Am. Chem. Soc. 142 13021 13029 10.1021/jacs.0c03880 32605368

[35] Kok K. Kuo C.L. Katzy R.E. Lelieveld L.T. Wu L. Roig-Zamboni V. et al 2022 1,6-*epi*-cyclophellitol cyclosulfamidate is a bona fide lysosomal α-glucosidase stabilizer for the treatment of pompe disease J. Am. Chem. Soc. 144 14819 14827 10.1021/jacs.2c05666 35917590 PMC9389588

[36] Lipsh-Sokolik R. Khersonsky O. Schröder S.P. Hoch S.Y. Davies G.J. Overkleeft H.S. et al 2023 Modularly designed protein fragments combine into thousands of active and structurally diverse enzymes Science. 379 195-201 10.1126/science.ade943 36634164

[37] Fenger T.H. Brumer H 2015 Synthesis and analysis of specific covalent inhibitors of endo-xyloglucanases Chembiochem 16 575 583 10.1002/cbic.201402663 25663665

[38] Janda K.D. Lo L.C. Lo C.H. Sim M.M. Wang R. Wong C.H. et al 1997 Chemical selection for catalysis in combinatorial antibody libraries Science 275 945 948 10.1126/science.275.5302.945 9020070

[39] Tsai C.S. Li Y.K. Lo L.C 2002 Design and synthesis of activity probes for glycosidases Org. Lett. 4 3607 3610 10.1021/ol0265315 12375899

[40] Lu C.P. Ren C.T. Lai Y.N. Wu S.H. Wang W.M. Chen J.Y . et al 2005 Design of a mechanism-based probe for neuraminidase to capture influenza viruses Angew. Chem. Int. Ed. 44 6888 6892 10.1002/anie.200501738 16215975

[41] Whidbey C. Sadler N.C. Nair R.N. Volk R.F. DeLeon A.J. Bramer L.M. et al 2019 A probe-enabled approach for the selective isolation and characterization of functionally active subpopulations in the gut microbiome J. Am. Chem. Soc. 141 42 47 10.1021/jacs.8b09668 30541282 PMC6533105

[42] Kwan D.H. Chen H.M. Ratananikom K. Hancock S.M. Watanabe Y. Kongsaeree P.T. et al 2011 Self‐immobilizing fluorogenic imaging agents of enzyme activity Angew. Chem. Int. Ed. 50 300 303 10.1002/anie.201005705 21184404

[43] Yariv J. Wilson K.J. Hildesheim J. Blumberg S 1971 Labelling of the active site of beta-galactosidase by N-bromoacetyl beta-D-galactopyranosylamine FEBS Lett. 15 24 26 10.1016/0014-5793(71)80070-4 11945805

[44] Jain N. Tamura K. Déjean G. Van Petegem F. Brumer H 2021 Orthogonal active-site labels for mixed-linkage endo-β-glucanases ACS Chem. Biol. 16 1968 1984 10.1021/acschembio.1c00063 33988963

[45] Chauvigné-Hines L.M. Anderson L.N. Weaver H.M. Brown J.N. Koech P.K. Nicora C.D. et al 2012 Suite of activity-based probes for cellulose-degrading enzymes J. Am. Chem. Soc. 134 20521 20532 10.1021/ja309790w 23176123 PMC3538167

[46] Schröder S.P. Boer C.d. McGregor N.G.S. Rowland R.J. Moroz O. Blagova E . et al 2019 Dynamic and functional profiling of xylan-degrading enzymes in *Aspergillus* secretomes using activity-based probes ACS Cent. Sci. 5 1067 1078 10.1021/acscentsci.9b00221 31263766 PMC6598175

[47] Simmons T.J. Mortimer J.C. Bernardinelli O.D. Poppler A.C. Brown S.P. deAzevedo E.R . et al 2016 Folding of xylan onto cellulose fibrils in plant cell walls revealed by solid-state NMR Nat. Commun. 7 13902 10.1038/ncomms13902 28000667 PMC5187587

[48] Rennie E.A. Scheller H.V 2014 Xylan biosynthesis Curr. Opin. Biotechnol. 26 100 107 10.1016/j.copbio.2013.11.013 24679265

[49] Schröder S.P. Offen W.A. Males A. Jin Y. Boer C.d. Enotarpi J . et al 2021 The development of non-hydrolysable oligosaccharide activity-based inactivators for endoglycanases: a case study on a-1,6 mannanases Chem. Eur. J. 27 9519 9523 10.1002/chem.202101255 33878235 PMC8362039

[50] Boer C.d. McGregor N.G.S. Peterse E. Schröder S.P. Florea B.I. Jiang J . et al 2020 Glycosylated cyclophellitol-derived activity-based probes and inhibitors for cellulases RSC Chem. Biol. 1 148 155 10.1039/d0cb00045k 34458755 PMC8341922

[51] McGregor N.G.S. Boer C.d. Santos M. Haon M. Navarro D. Schroder S . et al 2022 Activity-based protein profiling reveals dynamic substrate-specific cellulase secretion by saprotrophic basidiomycetes Biotechnol. Biofuels 15 6 10.1186/s13068-022-02107-z PMC8764865 35418096

[52] Scott C. McGregor N. Leadbeater D. Oates N. Hoßbach J. Abood A . et al 2024 *Parascedosporium putredinis* NO1 tailors its secretome for different lignocellulosic substrates Microbiol. Spectr. 12 e03943-03923 10.1128/spectrum.03943-23 38757984 PMC11218486

[53] McGregor N.G.S. Overkleeft H.S. Davies G.J 2022 Detecting and identifying glycoside hydrolases using cyclophellitol-derived activity-based probes Meth. Enzymol. 664 103 134 10.1016/bs.mie.2022.01.007 35331370

[54] Pauly M. Keegstra K 2016 Biosynthesis of the plant cell wall matrix polysaccharide xyloglucan Annu. Rev. Plant Biol. 67 235 259 10.1146/annurev-arplant-043015-112222 26927904

[55] McGregor N.G.S. de Boer C. Foucart Q.P.O. Beenakker T. Offen W.A. Codée J.D.C . et al 2023 A multiplexing activity-based protein profiling platform for dissection of a native bacterial xyloglucan-degrading system ACS Cent. Sci. 9 2306 2314 10.1021/acscentsci.3c00831 38161374 PMC10755729

[56] Kim S.J. Chandrasekar B. Rea A.C. Danhof L. Zemelis-Durfee S. Thrower N. et al 2020 The synthesis of xyloglucan, an abundant plant cell wall polysaccharide, requires CSLC function Proc. Natl. Acad. Sci. U.S.A. 117 20316 20324 10.1073/pnas.2007245117 32737163 PMC7443942

[57] Chen Y. Armstrong Z. Artola M. Florea B.I. Kuo C.L. de Boer, C. et al 2021 Activity-based protein profiling of retaining α-amylases in complex biological samples J. Am. Chem. Soc. 143 2423 2432 10.1021/jacs.0c13059 33497208 PMC7883350

[58] Pickles I.B. Chen Y. Moroz O. Brown H.A., de Boer, C. Armstrong Z. et al 2025 Precision Activity-Based α-Amylase Probes for Dissection and Annotation of Linear and Branched-Chain Starch-Degrading Enzymes Angew. Chem. Int. Ed. 64 e202415219 10.1002/anie.202415219 39601378

[59] Caner S. Zhang X. Jiang J. Chen H.M. Nguyen N.T. Overkleeft H . et al 2016 Glucosyl epi-cyclophellitol allows mechanism-based inactivation and structural analysis of human pancreatic α-amylase FEBS Lett. 590 1143 1151 10.1002/1873-3468.12143 27000970

[60] Tailford L.E. Offen W.A. Smith N.L. Dumon C. Morland C. Gratien J. et al 2008 Structural and biochemical evidence for a boat-like transition state in beta-mannosidases Nat. Chem. Biol. 4 306 312 10.1038/nchembio.81 18408714

[61] Ducros V.M.A. Zechel D.L. Murshudov G.N. Gilbert H.J. Szabo L. Stoll D . et al . Substrate distortion by a beta-mannanase: snapshots of the Michaelis and covalent-intermediate complexes suggest a B_2,5_ conformation for the transition state Angew. Chem. Int. Ed. 41 2824 2827 10.1002/1521-3773(20020802)41:153.0.co;2-g 12203498

[62] McGregor N.G.S. Kuo C.L. Beenakker T.J.M. Wong C.S. Offen W.A. Armstrong Z. et al 2022 Synthesis of broad-specificity activity-based probes for *exo* -β-mannosidases Org. Biomol. Chem. 20 877 886 10.1039/D1OB02287C 35015006 PMC8790593

[63] Wang W. Andric N. Sarch C. Silva B.T. Tenkanen M. Master E.R 2018 Constructing arabinofuranosidases for dual arabinoxylan debranching activity Biotechnol. Bioeng. 115 41 49 10.1002/bit.26445 28868788

[64] McGregor N.G.S. Artola M. Nin-Hill A. Linzel D. Haon M. Reijngoud J. et al 2020 a) Rational design of mechanism-based inhibitors and activity-based probes for the identification of retaining α-L-arabinofuranosidases J. Am. Chem. Soc. 142 4648 4662 10.1021/jacs.9b11351 32053363 PMC7068720

[65] McGregor N.G.S. Turkenburg J.P. Mørkeberg Krogh K.B.R. Nielsen J.E. Artola M. Stubbs K.A. et al 2020 Structure of a GH51 α-L-arabinofuranosidase from Meripilus giganteus: conserved substrate recognition from bacteria to fungi Acta Crystallogr. D. Struct. Biol. 76 1124 1133 10.1107/S205979832001253X 33135683 PMC7604909

[66] Goddard-Borger E.D. Williams S.J 2017 Sulfoquinovose in the biosphere: occurrence, metabolism and functions Biochem. J. 474 827 849 10.1042/BCJ20160508 28219973

[67] Kaur A. Pickles I.B. Sharma M. Madeido Soler N. Scott N.E. Pidot S.J. et al 2023 Widespread family of NAD^+^-dependent sulfoquinovosidases at the gateway to sulfoquinovose catabolism J. Am. Chem. Soc. 145 28216 28223 10.1021/jacs.3c11126 38100472 PMC10755693

[68] Speciale G. Jin Y. Davies G.J. Williams S.J. Goddard-Borger E.D 2016 YihQ is a sulfoquinovosidase that cleaves sulfoquinovosyl diacylglyceride sulfolipids Nat. Chem. Biol. 12 215 217 10.1038/nchembio.2023 26878550

[69] Li Z. Pickles I.B. Sharma M. Melling B. Pallasdies L. Codée J . et al 2024 Detection of sulfoquinovosidase activity in cell lysates using activity‐based probes Angew. Chem. Int. Ed. 63 e202401358 10.1002/anie.202401358 38647177

[70] Kocharova N.A. Knirel Y.A. Shashkov A.S. Kochetkov N.K. Pier G.B 1988 Structure of an extracellular cross-reactive polysaccharide from Pseudomonas aeruginosa immunotype 4 J. Biol. Chem. 263 11291 11295 10.1016/S0021-9258(18)37956-0 3136157

[71] Ruijgrok G. Offen W.A. Raju D. Patsos T. de Boer C. Wu L . et al 2025 Bespoke Activity-Based Probes Reveal that the Pseudomonas aeruginosa Endoglycosidase, PslG, Is an Endo-β-glucanase J. Am. Chem. Soc. 147 8578 8586 10.1021/jacs.4c16806 39999423 PMC11912335

[72] Zhou G. Rusnac D.V. Park H. Canzani D. Nguyen H.M. Stewart L. et al 2024 An artificial intelligence accelerated virtual screening platform for drug discovery Nat. Commun. 15 7761 10.1038/s41467-024-52061-7 39237523 PMC11377542

[73] Bains R.K. Nasseri S.A. Wardman J.F. Withers S.G 2024 Advances in the understanding and exploitation of carbohydrate-active enzymes Curr. Opin. Chem. Biol. 80 102457 10.1016/j.cbpa.2024.102457 38657391

